# Comparative mapping of quantitative trait loci for Fusarium head blight resistance and anther retention in the winter wheat population Capo × Arina

**DOI:** 10.1007/s00122-015-2527-8

**Published:** 2015-05-16

**Authors:** Maria Buerstmayr, Hermann Buerstmayr

**Affiliations:** Department for Agrobiotechnology Tulln, BOKU-University of Natural Resources and Life Sciences Vienna, Konrad Lorenz Str. 20, 3430 Tulln, Austria

## Abstract

*****Key message***:**

**Fusarium resistance in Arina is highly quantitative and governed by multiple small effect QTL. Anther retention has a high correlation with FHB susceptibility and appears a practicable indirect selection target for enhancing FHB resistance.**

**Abstract:**

The Swiss winter wheat cultivar Arina possesses a high resistance level constituted by a number of small to medium effect QTL reported from three independent mapping populations. Yet these overlap only for one resistance QTL on the long arm of chromosome 1B. The present study characterized Fusarium head blight (FHB) resistance in a population of 171 recombinant inbred lines from a cross between Arina (resistant) and Capo (moderately resistant). The population was evaluated for FHB resistance under field conditions for 3 years. Additionally, we phenotyped anther retention, plant height and flowering date to analyze their association with resistance. Lines with a low proportion of retained anthers after flowering and tall plants were significantly less diseased, while flowering date had no association with FHB severity. QTL analysis identified eight small to medium effect QTL for FHB severity, of which QTL on 1BS, 3B, 4AL and 6BL likely correspond to resistance alleles already detected in previously studied Arina populations. QTL for anther retention mapped to 4AL, 6BL and 5AS. Notably, QTL on 4AL and 6BL overlapped with QTL for FHB severity. A single small effect QTL for plant height was detected on 5AS and no QTL was identified for flowering date. Genotypes having three or four resistance alleles in combination showed a good resistance level, indicating pyramiding resistance QTL as a powerful approach for breeding resistant cultivars. Selection for rapid and complete anther extrusion appears promising as an indirect selection criterion for enhancing FHB resistance.

**Electronic supplementary material:**

The online version of this article (doi:10.1007/s00122-015-2527-8) contains supplementary material, which is available to authorized users.

## Introduction

Fusarium head blight (FHB) of wheat, predominantly caused by *Fusariumgraminearum* and *Fusarium culmorum,* is a serious threat for wheat production in temperate regions throughout the world (McMullen et al. 1997). This destructive disease leads to losses in yield and quality (Bai and Shaner 1994) and to contamination with mycotoxins. These mycotoxins are harmful for human and animal livestock and as a consequence render harvests unsuitable and unsafe for food and feed (Parry et al. 1995; Gilbert and Tekauz 2000). Resistance to FHB is complex, modulated by polygenes and environmental factors (Buerstmayr et al. 2009; Liu et al. 2009; Löffler et al. 2009). Several components of resistance have been described. Resistance to initial infection (type 1) and resistance to spread of the pathogen within the spike (type 2) were first described by Schroeder and Christensen (1963) and since then investigated in many FHB studies. Active and passive resistance mechanisms contribute to FHB resistance (Mesterhazy 1995). Active resistance factors depend on the physiological defense response of the host plant. Passive resistance factors include morphological features, which alter conditions for primary infection and fungal growth development. One prominent morphological trait is plant height. Shorter plants are more at risk for infection from splash-dispersed spores than taller plants (Jenkinson and Parry 1994). Wheat spikes at different height are exposed to different microclimatic conditions, for instance varying humidity and temperature, which influence FHB severity (Hilton et al. 1999; Yan et al. 2011). Plant height contributes thus to both primary infection and fungal development.

Wheat appears to be most sensitive during anthesis when warm and humid conditions promote fungal infection (Pugh et al. 1933; Parry et al. 1995). Floral traits as well as external environmental factors at the time of anthesis very likely influence FHB severity. Since Arthur (1891) first reported a positive effect of early maturing cultivars on FHB resistance, several authors confirmed this finding (Gervais et al. 2003; Paillard et al. 2004; Schmolke et al. 2005). Escapes from infection have been discussed as a possible cause for these findings. Buerstmayr et al. (2008) conducted a multi-environment evaluation of 56 diverse wheat genotypes to study the stability of FHB resistance. No systematic association between flowering date and FHB severity was found among these.

Arthur (1891) first recorded that FHB was a floral infection disease. Since then, several floral traits have been evaluated for their associations to FHB resistance. Investigating the infection biology of Fusarium made clear that infection occurs inside the floral cavity (Pugh et al. 1933; Kang and Buchenauer 2000; Zange et al. 2005). Hyphal networks are usually formed on the inner surface of lemma, glume and palea, but not on the outer surfaces. Pugh et al. (1933) stated that as long as flowers of spikelets remain closed, the spikelet appears to be effectively protected against infection. Accordingly, studies on narrow flowering and floral opening duration indicated a positive impact of narrow and short floral opening on FHB resistance (Gilsinger et al. 2005). Kubo et al. (2010, 2013a) found that cleistogamous (closed flowering) cultivars showed less initial FHB infection (enhanced type 1 resistance) than chasmogamous (open flowering) cultivars. A follow-up study included the extent of anther extrusion as an additional factor. FHB severity was lowest in closed flowering lines, followed by lines with full anther extrusion, while lines with partially exposed anthers were most sensitive to FHB (Kubo et al. 2013b). All studies which investigated the association between the extent of anther extrusion and FHB resistance so far observed an increase of infection in the presence of retained anthers (Dickson et al. 1921; Tu 1953; Graham and Browne 2009; Skinnes et al. 2010; Lu et al. 2013; He et al. 2014; Kubo et al. 2013a, b). These results correspond with the observations that emasculation of anthers reduced FHB infection (Liang et al. 1981). Even though anthers are not necessarily required for a successful infection (Tu 1930; Schroeder 1955; Kang and Buchenauer 2000), the fungus exhibits a special affinity to pollen and anthers (Miller et al. 2004). Pugh et al. (1933) noted in microscopic studies of early infection that a number of spikelets developed mycelium solely within retained anther tissue. Kang and Buchenauer (2000) observed inside the floral cavity a stronger hyphal growth of the fungus on anthers than on the inner surface of the lemma or palea. All these observations suggest that retained anthers promote hyphal growth. Strange and Smith (1971) and Strange et al. (1974) suspected the presence of choline and betaine in anthers as important fungal growth stimulants. However, subsequent studies found no effects of either substance on fungal growth (Engle et al. 2004). Given that anthers are an easy and fast decaying tissue, they potentially offer little resistance to the fungus and thus constitute a preferred target for initial infection. So far only two QTL mapping studies (Skinnes et al. 2010; Lu et al. 2013) assessed the association between FHB traits (FHB severity, DON content) and the extent of anther extrusion (AE). Both revealed significant correlations between AE and FHB traits. Although four QTL for anther retention were found by Skinnes et al. (2010), these overlapped at only one location with a FHB resistance QTL. Lu et al. (2013) found five QTL for anther extrusion, of which each co-located with QTL for FHB resistance after spray or grain-spawn inoculation indicating a pleiotropic effect of anther extrusion on type 1 FHB resistance.

Mesterhazy (1995) suspected awns as a possible component for increased susceptibility. The larger surface of awned spikes makes infection by airborne conidia more likely and dew caught by the awns provide potentially more humid conditions for successful infection.

The aim of the current study was to characterize the genetics of FHB resistance and potentially associated morphological traits, such as plant height, anther retention, awnedness and date of anthesis through molecular mapping. For this purpose, a recombinant inbred line (RIL) population was developed from a cross between the Austrian and Swiss winter wheat cultivars Capo and Arina. Results will be compared to previous studies analyzing the RIL population Arina × Forno (Paillard et al. 2004), the double haploid (DH) population Arina × Riband (Draeger et al. 2007) and the DH population Arina × NK93604 (Semagn et al. 2007).

## Materials and methods

### Plant material

A mapping population of 171 F_5_ derived F_7_ (F_5:7_) recombinant inbred lines (RILs) was developed by single seed decent from a cross of Capo and Arina. Capo (Diplomat/Purdue5517//Extrem/HP3517) is a winter wheat cultivar developed by Probstdorfer Saatzucht, Austria. It is moderately resistant to FHB and has an awned spike phenotype. Arina (Moisson/Zenith) is a winter wheat cultivar developed by Agroscope, Switzerland. Arina is an awnless cultivar and has a medium to high resistance level to FHB (Paillard et al. 2004; Mesterhazy et al. 1999).

### Field experiments

The RIL population, the parental lines and several control lines were tested in field experiments at IFA-Tulln, Austria (16^o^04,16′E, 48^o^19,08′N, 177 m above sea level) during three consecutive years (2011, 2012, 2013). Experiments were arranged as randomized complete block design with two blocks. Plots consisted of double rows of 1 m length with 17 cm spacing. Sowing time was late autumn in all years. The two blocks of each experiment were purposely sown 2–3 weeks apart depending on the specific weather conditions, which delayed anthesis by 1–3 days for the later-sown blocks. Seed treatment, sowing density and crop management were essentially the same as described by Buerstmayr et al. (2002). Spray inoculations were performed individually when 50 % of the heads within a plot were flowering, and repeated two days later. Hundred ml of a macroconidial suspension with a spore concentration of 2.5 × 10^4^ ml^−1^ was sprayed onto the heads using a motor-driven back-pack sprayer in the late afternoon. An automated mist-irrigation system provided humidity for 20 h after inoculation to facilitate infection. A macroconidial suspension of the *F. culmorum* single-spore isolate ‘Fc91015’ was used for inoculation, which was prepared as described by Buerstmayr et al. (2000). The percentage of infected spikelets per plot was estimated visually 10, 14, 18, 22 and 26 days post the first inoculation (dpi). Scheduling FHB severity scoring relative to anthesis date avoids confounding FHB severity with earliness. This inoculation and scoring method reflects overall FHB resistance and provides combined information on type 1 and type 2 resistances. The area under the disease progress curve (AUDPC) was calculated as described by Buerstmayr et al. (2002) and used as an integrated measure of FHB severity. Date of flowering was recorded for each plot and converted into number of days after May 1st. Anther retention (AR) was inspected on 20 florets per plot (four basal florets in the middle of the spike on five randomly chosen heads per plot 5 days after anthesis) on all entries of years 2012 and 2013. Individual florets were opened manually and inspected for retained anthers. AR was expressed in % florets with at least one anther remaining inside the floret or trapped between lemma and palea. In contrast to visually estimating anther extrusion, the data based on counting retained anthers are neither impaired by heavy rainfall or strong wind, nor by differences between genotypes in their speed and amount of throwing off extruded anthers. Plant height (to the top of the head excluding awns) was measured in cm in each trial. RILs were classified as awned, awnless or heterogeneous for this trait. This trait was treated as a morphological marker.

### Statistical analysis of field experiments

Analysis of variance (ANOVA) and correlation of field data were calculated in SAS/STAT version 9.3 (SAS Institute Inc. 2008). Distributions of residuals were tested for normality using PROC UNIVARIATE applying the Shapiro–Wilk statistics, and homogeneity of variances was verified by Levene’s test. A square root transformation was performed for the FHB severity data (measured in AUDPC) to achieve normality. Statistical analyses were carried out with the transformed AUDPC data. PROC MIXED procedure was used to calculate the ANOVA. Genotypes were treated as fixed effects and year, block within year and genotype-by-year interaction as random effects. Models were modified leaving one random variable out at a time. Models were compared by AIC (Akaike information criterion) criterion (Akaike 1974) and the best model was used to estimate the variance components. Variance components were determined by the restricted maximum likelihood (REML) method. Heritability coefficients were estimated from variance components with the equation $H = \sigma_{\text{G}}^{ 2} /(\sigma_{\text{G}}^{ 2} + \sigma_{\text{GxY}}^{ 2} /y + \sigma_{\text{E}}^{ 2} /yr)$, where $\sigma_{\text{G}}^{ 2}$ genotypic variance, $\sigma_{\text{GxY}}^{ 2}$ genotype-by-year interaction variance, $\sigma_{\text{E}}^{ 2}$ residual variance, *y* number of years, and *r* number of replications (Nyquist 1991). For the estimation of the heritability coefficients, all effects were considered random. Spearman rank-correlation coefficients of individual traits were estimated for all pairwise experiment combinations and correlations between FHB severity, plant height, flowering date and anther retention were estimated for line means within each experiment and for means over all experiments.

### Molecular marker analysis and map construction

Molecular marker analysis and map construction of population Capo × Arina are described in Buerstmayr et al. (2014). Lines were additionally genotyped with SSR markers *Barc24*, *Barc70*, *Gwm558*, *Wmc382* and *Wmc389*.

### QTL analysis

QTL calculations were carried out with R version 3.0.2 (R Development Core Team 2011) using the R/qtl package 1.28-19–12 (Broman et al. 2003). Missing genotypic information was imputed using the multiple imputation method of Sen and Churchill (2001). Genome-wide QTL searches were conducted separately for each experiment and the overall means across all experiments. Interval mapping was performed using a single QTL genome scan and pairwise epistatic QTL interactions were calculated using a two-dimensional QTL scan via Haley–Knott regression (Haley and Knott 1992). LOD significance thresholds of the respective trait for type I error rates of *α* < 0.1, *α* < 0.05 and *α* < 0.01 were determined by running 1000 permutations on the single- and two-dimensional QTL scan. Finally, a multiple QTL mapping (MQM) analysis was performed on the individual experiments and on the overall means across experiments. Individual MQM models were fitted including experiment-specific significant QTL determined by the single- and two-dimensional QTL scan. The overall fit of the full model against the null model was tested by ANOVA and the estimated additive effect and the percentage of phenotypic variance explained by each QTL were obtained from the MQM analysis. The QTL support interval criterion was defined by a LOD decrease of 1.5 from the maximum LOD position. Linkage groups, LOD bars and LOD profiles of QTL were drawn with MapChart v2.2 (Voorrips 2002). RILs were grouped according to their genotypic information that means by the number of resistance improving QTL alleles determined by the MQM model for the overall mean FHB severity. Differences between the means of these groups were compared using the Tukey multiple range test.

## Results

### Trait variation and trait correlations

**Fig. 1:**
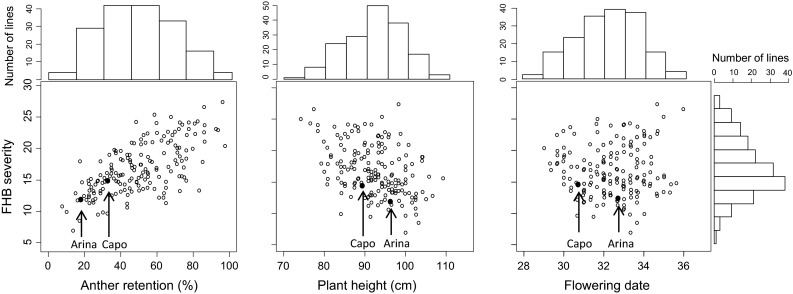
Scatterplots of overall means for FHB severity against anther retention (%), plant height (cm) and flowering date (days after May 1st) with marginal histograms of their frequency distribution. FHB severity is based on overall mean values of the square root transformed AUDPC data. *Arrows* and *filled dots* indicate the positions of the parents

The population displayed continuous variation for FHB severity, anther retention, plant height and date of flowering (Fig. 1). The means of the parental lines, the means and ranges of the RIL population for the individual experiments as well as for the averaged mean across all experiments for FHB severity, plant height, AR and flowering date are reported in Table 1. Online resource 1 summarizes population statistics for %FHB diseased spikelets 26 dpi, AUDPC and AUDPC-transformed. Transformed AUDPC data were used as measure for FHB severity in this study, while %FHB diseased spikelets and AUDPC (untransformed) are not shown in the subsequent statistical analyses presented here. In all experiments, Arina was less diseased than Capo, although this difference was not significant in 2012. Arina was on average 6 cm taller, reached anthesis 2 days later, and kept fewer anthers inside the spikelets after flowering compared to Capo (Table 1). Transgressive segregation for FHB severity and for anther retention was observed in all experiments. Spearman correlation coefficients between traits and between experiments are provided in Online resource 2. Correlations between experiments were highly significant and ranged from *r* = 0.60 to 73 for FHB severity, from *r* = 0.79 to 0.84 for plant height, from *r* = 0.73 to 0.80 for anthesis and was *r* = 0.77 for anther retention. Anther retention was positively correlated with FHB severity (*r* = 0.63) reflecting the lower FHB severity on plants with high anther extrusion. All experiments showed highly significant negative correlations between FHB severity and plant height (*r* = −0.39), with taller plants being more resistant. Flowering date had no influence on FHB severity. The AIC criterion determined the model including years, blocks within year and the genotype-by-year interaction as random effects as the best model. ANOVA revealed highly significant variation due to genotypes for all determined traits. Generally, $\sigma_{{\rm Genotype} \, \times \,  {\rm Year}}^{ 2}$ variance components were low compared to $\sigma_{\text{Year}}^{ 2}$ for all traits (Table 2). Heritability coefficients for FHB severity, anther retention, plant height and flowering date were 0.86, 0.87, 0.93 and 0.91, respectively.

**Table 1:** Means of parents and population, minimum and maximum values of population, least significant differences at *α* < 0.05 (LSD) and broad-sense heritability coefficient (*H*) or repeatability of analyzed traits

Trait	Experiment	Parents		Population				
		Arina	Capo	Mean	Min	Max	LSD5 %	*H*
FHB severity^a^	2011	10.9	13.5	16.0	5.9	26.4	1.97	0.86^b^
	2012	12.8	15.0	14.7	5.1	26.6	2.84	0.72^b^
	2013	12.4	16.2	19.9	7.6	33.1	3.56	0.69^b^
	Overall mean	12.0	14.9	16.8	7.0	27.4	2.84	0.86
Anther retention (%)	2012	20.0	22.5	55.0	7.5	100.0	12.59	0.78^b^
	2013	15.0	32.5	45.4	2.5	100.0	9.12	0.87^b^
	Overall mean	17.5	27.5	50.3	7.5	97.5	11.03	0.87
Plant height (cm)	2011	102.5	92.5	100.6	77.5	115.0	3.47	0.86^b^
	2012	95.0	87.5	88.6	67.5	110.0	3.51	0.86^b^
	2013	90.0	87.5	87.6	70.0	105.0	3.20	0.86^b^
	Overall mean	95.8	89.2	92.3	74.2	109.2	3.40	0.93
Flowering date (days after May 1st)	2011	28.0	25.0	27.2	25.0	31.0	0.85	0.81^b^
	2012	30.0	28.0	29.2	26.0	33.0	0.85	0.80^b^
	2013	40.0	39.0	41.0	36.0	45.0	0.82	0.90^b^
	Overall mean	32.7	30.7	32.4	27.0	36.0	0.87	0.91

^a^Calculated from the transformed AUDPC data

^b^Repeatability

**Table 2:** Variance component estimates of year $\sigma_{\text{Year}}^{ 2}$, block within year $\sigma_{\text{Block within Year}}^{ 2}$, genotype × year $\sigma_{{{\text{Genotype}}\, \times \, {\text{Year}}}}^{2}$ and the residual effects $\sigma_{\text{error}}^{ 2}$ for FHB severity, plant height, anther retention (%) and flowering date

Trait	Variance component			
	σ_Year_^2^	σ_Block within Year_^2^	σ_Genotype × Year_^2^	σ_error_^2^
FHB severity^a,c^	4.65	5.47	1.57	10.53
Plant height^c^	51.59	0.27	2.66	14.89
Anther retention %^d^	37.47	8.63	26.60	157.40
Flowering date^b,c^	55.52	1.70	0.09	0.97

^a^Calculated from the transformed AUDPC data

^b^Flowering date in days after May 1st

^c^173 Genotypes, 3 years, two blocks within year

^d^173 Genotypes, 2 years, two blocks within year

### Linkage group

Molecular marker analysis and map construction of population Capo x Arina are described in Buerstmayr et al. (2014). In total, 437 unique marker loci, consisting of DArT and SSR markers, were used for map construction. The map length was 1644 cM, with an average distance of 3.8 cM. Markers fell into 34 linkage groups covering 635 cM on genome A, 727 cM on genome B and 256 cM on genome D. Twenty-six cM could not be unambiguously assigned to a chromosome; no linkage group could be attributed to chromosome 4D.

### QTL analysis

#### QTL for Fusarium head blight

**Fig. 2:**
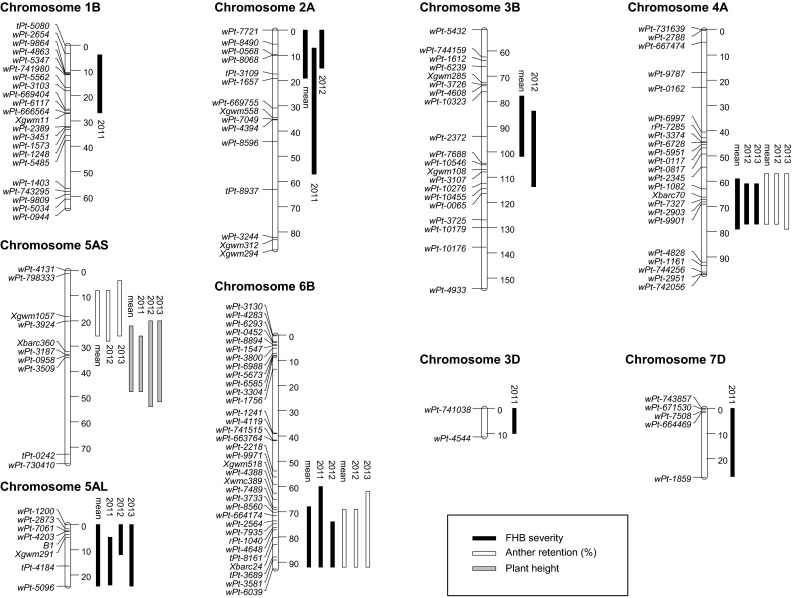
Linkage maps and positions of QTL for FHB severity, anther retention (%) and plant height (cm) determined by the MQM model. FHB severity is based on the transformed AUDPC data. Bars of the QTL support interval for the respective experiments and trait are on the *right*. Bar size indicates an LOD decrease of 1.5 from the maximum LOD value

**Fig. 3:**
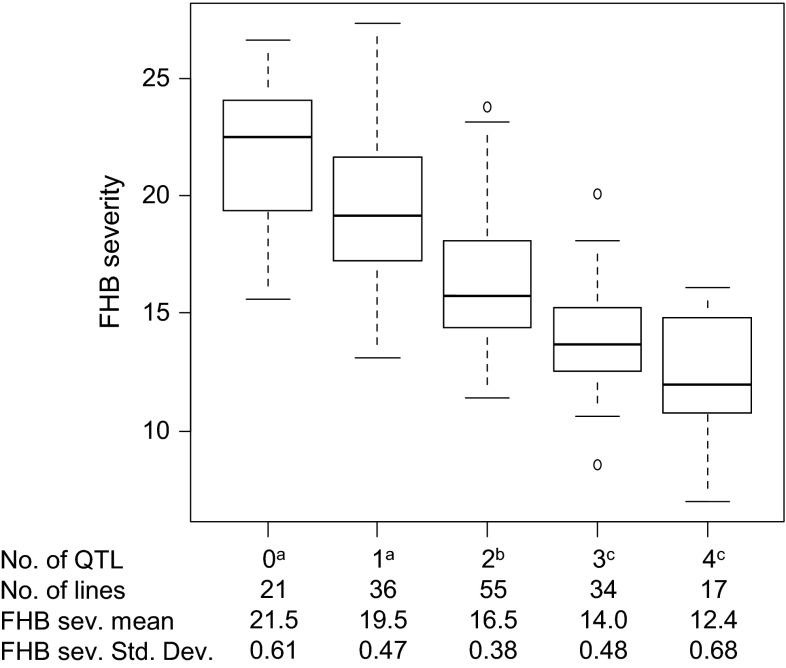
Boxplots of groups of lines with increasing number of FHB resistance improving alleles. Number of QTL classes is based on the MQM analysis for the overall means across experiments using the square root transformed AUDPC data as measure for FHB severity. Medians are indicated by *solid lines*. QTL groups with different index letters are significantly different based on a Tukey test at *p* < 0.05

MQM analysis identified eight different QTL for FHB severity, which mapped to chromosomes 1BS, 2AS, 3B, 3D, 4AL, 5AL, 6BL and 7D. Results of the QTL mapping are summarized in Table 3. Linkage groups and confidence ranges of QTL are shown in Fig. 2. Online resource 3 provides linkage groups and LOD profiles of QTL detected in the Capo/Arina population. Online resource 3 additionally indicates approximate positions of QTL identified in the populations Arina/Forno (Paillard et al. 2004), Arina/Riband (Draeger et al. 2007) and Arina/NK93604 (Semagn et al. 2007). The effects of individual QTL were generally low and contributed between 4.3–8.6 % to the phenotypic variance (Table 3). The favorable allele originated from Arina apart from QTL on 5AL and 3D. The QTL on 5AL was consistently detected in all three individual experiments and for the mean across all experiments. Notably, the *B1* gene modulating awnedness is located within its confidence interval and it was the allele from the awned cultivar Capo which improved resistance. QTL effects on 2AS and 6BL were significant in 2011, 2012 and for the overall mean. The QTL on 6BL mapped within a 25 cM interval placing the peak marker close to *Xbarc24*. QTL location on 2AS was relatively unsteady, placing the position of the QTL to the distal end of 2AS in experiment 2012 and the overall mean, while it appeared more proximal in 2011. The effect of the QTL on 4AL was significant in 2012, 2013 and the analysis with the overall means, its confidence interval stretched over 20 cM and reached its maximum LOD value at marker *wPt*-*2903*. The QTL on 3B was found in the analyses of the individual year 2012 and with the overall mean. QTL on 1BS, 3D and 7D were identified in 2011 only. Across all experiments, the total phenotypic variance explained by the discovered QTL was 40 %. QTL for FHB severity on 4AL and 6BL coincided with the QTL for AR, and the Arina allele reduced both AR and FHB severity. Notably, for the FHB QTL on 4AL and 6BL, equally high or slightly higher LOD values were obtained for FHB severity at early scoring dates (% diseased spikelets at 14 and 18 dpi), while QTL not associated with AR, apart from the QTL on 5AL, were highest at the latest scoring date (26 dpi) and for transformed AUDPC values (details not shown). No epistatic QTL interactions were revealed, thus QTL acted in an additive manner. Boxplots of Fig. 3 demonstrate continuously increasing resistance due to combining an increasing number of resistance conferring QTL alleles, although differences were not significant between groups of lines possessing either none or one resistance improving allele as well as between groups with three or four resistance improving alleles in combination.

**Table 3:** Locations and estimates of QTL for FHB severity, anther retention (%) and plant height using multiple QTL mapping

Trait	Experiment	Chromosome	Upper marker	Closest marker	Lower marker	LOD	PV %^a^	Add^b^
FHB severity^c^	2011	1BS	*tPt*-*5080*	*wPt*-*3103*	*wPt*-*6117*	4.6	5.6	−1.00
	2011	2AS	*wPt*-*8490*	*tPt*-*3109*	*tPt*-*8937*	4.0	6.5	−1.08
	2012	2AS	*wPt*-*7721*	*wPt*-*7721*	*tPt*-*3109*	5.2	8.3	−1.30
	Overall mean	2AS	*wPt*-*7721*	*wPt*-*7721*	*tPt*-*3109*	4.5	7.6	−1.13
	2012	3B	*wPt*-*10323*	*wPt*-*2372*	*wPt*-*0065*	4.1	6.4	−1.13
	Overall mean	3B	*wPt*-*10323*	*wPt*-*2372*	*wPt*-*7688*	3.1	5.0	−0.92
	2011	3D	*wPt*-*741038*	*wPt*-*741038*	*wPt*-*4544*	4.3	7.1	1.13
	2012	4AL	*wPt*-*2345*	*wPt*-*2903*	*wPt*-*4828*	4.8	7.6	−1.25
	2013	4AL	*wPt*-*2345*	*wPt*-*2903*	*wPt*-*4828*	3.5	8.6	−1.51
	Overall mean	4AL	*wPt*-*2345*	*wPt*-*2903*	*wPt*-*4828*	4.3	7.3	−1.13
	2011	5AL	*Xgwm291*	*wPt*-*5096*	*wPt*-*5096*	3.4	5.5	0.98
	2012	5AL	*wPt*-*1200*	*Xgwm291*	*tPt*-*4184*	4.3	6.8	1.16
	2013	5AL	*wPt*-*1200*	*tPt*-*4184*	*wPt*-*5096*	2.9	6.9	1.44
	Overall mean	5AL	*wPt*-*1200*	*Xgwm291*	*wPt*-*5096*	3.8	6.3	1.03
	2011	6BL	*Xgwm518*	*Xbarc24*	*wPt*-*6039*	4.6	4.3	−1.18
	2012	6BL	*Xwmc389*	*Xbarc24*	*wPt*-*6039*	3.5	5.4	−1.10
	Overall mean	6BL	*Xwmc389*	*tPt*-*3689*	*wPt*-*6039*	3.2	5.3	−1.01
	2011	7D	*wPt*-*743857*	*wPt*-*1859*	*wPt*-*1859*	5.0	8.3	−1.29
Anther retention (%)	2012	4AL	*wPt*-*2345*	*wPt*-*2903*	*wPt*-*4828*	5.9	10.9	−0.07
	2013	4AL	*wPt*-*2345*	*wPt*-*2903*	*wPt*-*4828*	5.7	11.1	−0.07
	Overall mean	4AL	*wPt*-*2345*	*wPt*-*1082*	*wPt*-*4828*	7.3	12.8	−0.07
	2012	5AS	*wPt*-*798333*	*wPt*-*3924*	*Xbarc360*	3.7	6.5	0.06
	2013	5AS	*wPt*-*798333*	*wPt*-*3924*	*Xbarc360*	2.9	5.5	0.05
	Overall mean	5AS	*wPt*-*798333*	*wPt*-*3924*	*Xbarc360*	4.2	7.1	0.06
	2012	6BL	*wPt*-*3733*	*wPt*-*4648*	*wPt*-*6039*	6.1	11.2	−0.08
	2013	6BL	*Xwmc389*	*wPt*-*4648*	*wPt*-*6039*	4.9	9.5	−0.07
	Overall mean	6BL	*wPt*-*3733*	*wPt*-*4648*	*wPt*-*6039*	6.4	11.3	−0.07
Plant height (cm)	2011	5AS	*wPt*-*3924*	*wPt*-*3187*	*tPt*-*0242*	4.9	12.5	2.82
	2012	5AS	*wPt*-*3924*	*wPt*-*3187*	*tPt*-*0242*	3.2	8.2	2.22
	2013	5AS	*wPt*-*3924*	*wPt*-*3187*	*tPt*-*0242*	2.9	7.8	1.99
	Overall mean	5AS	*wPt*-*3924*	*wPt*-*3187*	*tPt*-*0242*	4.5	11.4	2.39

^a^Percentage of phenotypic variance explained by the QTL

^b^Positive additive effects denote trait-increasing effect of the Arina allele; additive effects were estimated as half the difference between phenotype averages for the homozygote

^c^FHB severity results are based on the transformed AUDPC data

#### QTL for anther retention and plant height

QTL for AR were located on chromosomes 4A, 5A and 6B (Table 3; Fig. 2, Online resource 3). These QTL were consistently identified in all individual experiments and in the overall mean. The percentage of explained phenotypic variance ranged from 10 to 13 % for QTL on 4AL and 6BL and from 5 to 7 % for the QTL on 5AS. All three QTL together contributed 41 % to the total phenotypic variance of AR employing the overall mean across years. The QTL on 4AL is located between *wPt*-*2345* and *wPt*-*4828* and achieved the highest LOD value close to marker *wPt*-*2903*. The QTL support interval on 6BL covered a distance of 25 cM and was flanked by marker *Xwmc389* and *wPt*-*6039.* The Capo allele reduced AR on chromosome 5AS. QTL support interval on 5AS spanned over 20 cM with *wPt*-*3924* as peak marker. A QTL for plant height mapped close to the centromere of 5A and marginally overlapped with QTL for AR. The Capo allele was associated with reduced plant height. No significant QTL for flowering date were found.

## Discussion

The RILs of the population had a clear quantitative, continuous distribution with transgressive segregations towards higher and lower values for FHB severity, AR, plant height and flowering date (Fig. 1; Table 1). This suggests the involvement of several moderate to small effect QTL. Accordingly, eight QTL for FHB severity and three QTL for AR were identified (Table 3; Fig. 2, Online resource 3). A single small effect QTL was detected for plant height and no QTL were detected for flowering date, though heritabilities were higher than for FHB severity and AR. Apparently, no major genes are involved in modulating plant height and flowering date in this population, and the observed high heritability rests upon many genetic factors with individual contributions too small to surpass a significance threshold. Transgressive segregation towards lower FHB severity and higher AR indicated that both parents contributed favorable alleles, yielding beneficial gene combinations for some RILs.

### QTL for Fusarium head blight

The resistance to Fusarium head blight of Arina was studied previously in three independent populations (Paillard et al. 2004; Draeger et al. 2007; Semagn et al. 2007). These studies reported varying numbers of QTL. Eight, six and two QTL with resistance alleles derived from Arina were identified in the Arina/Forno, Arina/Riband and Arina/NK93604 populations, respectively, but only minimal overlaps were found between these three mapping populations. QTL identified in the present study as well as in the Arina/Forno and Arina/Riband populations were of minor to medium effect, observed in a subset of the conducted experiments or in single experiments only, while QTL reported in the Arina/NK93604 population appeared to be of major effect and stable across environments.

### Comparing QTL results of Capo/Arina, Arina/Riband, Arina/Forno and Arina/NK93604 population

Comparing marker arrangement and QTL positions of the Capo/Arina, Arina/Riband, Arina/Forno and Arina/NK93604 populations is non-trivial because only a small number of common markers exist across these populations. When comparing marker positions to consensus maps published by Somers et al. (2004), Crossa et al. (2007) and Marone et al. (2012), it became evident that five out of eight QTL identified for Fusarium resistance in the present study showed overlaps of their confidence intervals with QTL already reported in at least one of the previously studied Arina populations. Approximate chromosomal positions of these QTL with improved FHB resistance conferred by the Arina allele are, therefore, included in the Online resource 3. *Xbarc70* is in close proximity to the QTL peak on 4AL (Fig. 2, Online resource 3). *Xbarc70* was placed 8 cm proximal to *Xgwm160* in the consensus map of Somers et al. (2004). *Xgwm160* determines the distal end of the 4AL QTL interval in the Arina/Forno population. Accordingly, the QTL interval identified in Capo/Arina coincides with the QTL in the Arina/Forno population (Online resource 3). *Xbarc79* in the Arina/Riband population and *wPt*-*3581* of the current study both mapped close to the QTL peak on 6BL. These two markers mapped within 4 cM in the consensus map developed by Marone et al. (2012). Thus, QTL interval on 6BL in Arina/Riband and Capo/Arina population clearly overlaps (Fig. 2, Online resource 3). *Xbarc119* and *S12M13* were associated with AUDPC in Arina/Riband population. These markers are located within the confidence interval of the 1BS QTL identified in the present study, and QTL on 3B (centromeric) and 5AL mapped to corresponding intervals of the Arina/Forno population (Online resource 3). Interestingly, in our study it was the Capo allele which improved resistance on 5AL. Accordingly, QTL on 4AL, 6BL, 3B and 1BS likely refer to resistance alleles already reported by Draeger et al. (2007) or Paillard et al. (2004). QTL on 2AS mapped to an interval which was not associated with FHB resistance in any of the previously studied Arina populations. Location of QTL on 7D and 3D was not comparable between studies as no consensus markers were available. Although a QTL on 1BL was so far the only consensus QTL among Arina/Riband, Arina/Forno and Arina/NK93604, it was not identified in the present study. In summary, only part of the FHB resistance can be attributed to putatively identical resistance QTL among individual populations. Interestingly, there is no common resistance QTL allele across all four populations. Fusarium resistance of Arina is very complex and regulated by many different genomic regions with primarily small to moderate effect QTL. While a single QTL may not be enough to achieve the desired resistance level, a combination of three and more QTL is a suitable strategy to combat FHB.

### QTL for anther retention

AR exhibited continuous quantitative variation. Although heritability was very high, no more than 40 % of the total variance could be explained by QTL. Analogous observations were made by Skinnes et al. (2010) and Lu et al. (2013). Both found that a great part of the variation remained unexplained by QTL despite high heritability. In the present study, QTL were consistent and mapped to chromosomes 4AL, 5AS and 6BL (Table 3; Fig. 2, Online resource 3). All QTL identified were different from those reported by Skinnes et al. (2010) and Lu et al. (2013). The lack of coinciding QTL for AR among Arina/NK93604 and Capo/Arina population points towards a strong dependence of AR on the genetic background. Apparently, genetics of partial AR and its complement anther extrusion (AE) is complex. Different floral traits, for instance openness of florets, duration of flower opening, length of anthers and filaments, tenacity and form of glumes, may influence the extent of anther extrusion. High positive correlation was observed for AE to anther length and to duration of floral opening (Singh et al. 2007) and between duration of floral opening and flower opening width (Gilsinger et al. 2005). High heritability estimates were found for duration of floral opening, openness of florets (Atashi-Rang and Lucken 1978; Singh et al. 2007), anther length (Singh et al. 2007) and AE (Singh et al. 2007; Lu et al. 2013; Skinnes et al. 2010). Of these traits, AE is comparably simple and fast to quantify and, therefore, a recommended selection target to complement breeding for FHB resistance. Indeed, Taylor (2004) reported about preselection of early generation breeding material for high anther extrusion as a supportive indirect trait for FHB resistance even in years with low Fusarium disease pressure. High AE has no detrimental effect on agronomic traits. Regarding the renewed interest on hybrid wheat breeding, complete anther extrusion in the male parent is highly desired for improved cross-pollination. Flowering biology seems to predominantly affect primary infection, which refers to type 1 resistance and early FHB development. Thus, combining high anther extrusion with type 2 resistance QTL seems advisable.

### Association between Fusarium head blight and anther retention

The high correlation between FHB severity and AR is in good agreement with previous studies (Singh et al. 2007; Skinnes et al. 2010; Lu et al. 2013). This strong phenotypic correlation underlines the negative effect of retained anthers on Fusarium resistance. This dependency was reported early (Pugh et al.^1933^) and confirmed in a number of studies (Graham and Browne 2009; Skinnes et al. 2010; Lu et al. 2013; He et al. 2014; Kubo et al. 2013a, b). All QTL identified for AE were associated with FHB traits in the population Shanghai-3/Catbird x Naxos (Lu et al. 2013), while only one QTL for AE overlapped with FHB QTL in the Arina/NK93604 population (Skinnes et al. 2010). In the current study, two out of three AR QTL coincided with FHB QTL. Our results strongly support the conclusion made by Lu et al. (2013) that several of the FHB resistance QTL reported previously in numerous papers could actually be caused by AE. Conditions inside the floral cavity are much more favorable for the pathogen than those on the outer surface (Pugh et al. 1933; Kang and Buchenauer 2000) and infection usually occurs on the inner surfaces of lemmas and paleae (Zange et al. 2005). As a consequence, cleistogamy or narrow flowering might act as passive resistance factor. Kubo et al. (2010) found that cleistogamous RILs showed less initial FHB infection and Gilsinger et al. (2005) observed that narrow flower opening, which was associated with a short flowering time, reduced FHB incidence. Retained anthers and pollen are major targets during the initial stages of infection (Miller et al. 2004). Rapid and complete anther extrusion can, therefore, impede infection to some extent. Both cleistogamous, closed flowering wheat genotypes and genotypes with a high level of anther extrusion were less diseased than lines with partially extruded anthers (Pugh et al.^1933^; Liang et al. 1981; Kubo et al. 2010, 2013a, b; Lu et al. 2013; Skinnes et al. 2010; Graham and Browne 2009). While cleistogamous flowering reduces the chance of infection, full anther extrusion would decrease favorable conditions for growth of the fungus.

### Association of Fusarium resistance to plant height, date of flowering and awnedness

A significant negative correlation was observed between plant height and FHB severity, but the extent of the correlation was much smaller than the correlation between AR and FHB severity. Numerous publications reported a positive effect of increased plant height on reduced FHB severity when tested in field experiments. Many of these studies found coinciding QTL for plant height and FHB-related traits, often but not always, associated with large effect plant height genes (Gervais et al. 2003; Paillard et al. 2004; Steiner et al. 2004; Schmolke et al. 2005; Draeger et al. 2007; Voss et al. 2008; Srinivasachary et al. 2008, 2009; Häberle et al. 2009; Mao et al. 2010; Buerstmayr et al. 2011, 2012; Lu et al. 2013). In the present study, only one plant height QTL was detected which mapped close to the centromere of 5A. It partially overlapped with a QTL for AR, but was not associated with FHB severity. This QTL interval for plant height coincided with a plant height QTL identified in the Arina/Forno population. In both, Arina/Forno and the Arina/Riband populations, five plant height QTL were identified, but none co-located with the QTL in the respective other population. No information is available on plant height in the Arina/NK93604 population. Taller plants have a higher chance to escape infection and, therefore, contribute to type 1 resistance. Furthermore, increased height reduces the relative humidity around the spikes, which leads to less favorable microclimates for disease development. However, breeding for increased plant height is undesired and inapplicable because medium to short cultivars are required for modern crop management practices.

ANOVA revealed high heritability for flowering date. Despite this, no major QTL was detected. Involved genes may have remained hidden either due to an incomplete coverage of the genome, due to many small effect genes and/or non-additive gene action. In all 3 years of investigations, there was no association between flowering date and FHB resistance.

Mesterhazy (1995) hypothesized that awns may increase the risk of ear infection by Fusarium. A constant but small effect QTL for FHB overlapped with the gene for awnedness in 5AL, with the resistance allele derived by the awned cultivar Capo. Thus, results of the present study do not support the aforementioned hypothesis.

## Conclusions

Breeding for Fusarium head blight resistance is a continuous and challenging effort. Regionally adapted sources of resistance, such as the cultivar Arina for central Europe, can be readily integrated in modern winter wheat breeding programs. FHB resistance in this source is governed by multiple small effect genes. Lines with multiple resistance conferring alleles stacked together show a good level of FHB resistance. The extent of retained anthers after flowering is a highly heritable trait and correlated with increased FHB severity. QTL for FHB resistance and the extent of anther extrusion partly overlap. Selection of lines which quickly and completely extrude anthers after flowering leads to a highly correlated selection response towards increased FHB resistance under field conditions.

### Author contribution statement

Both authors are contributed equally to the field data observations, statistical data analysis, QTL analysis and manuscript writing.

## Electronic supplementary material

Supplementary material 1 (PDF 97 kb)

Supplementary material 2 (PDF 35 kb)

Supplementary material 3 (PDF 123 kb)
