# Supplementary material for: Comparative mapping of quantitative trait loci for Fusarium head blight resistance and anther retention in the winter wheat population Capo × Arina
Source: Theor Appl Genet. 2015 May 16;128(8):1519–30. doi: 10.1007/s00122-015-2527-8 (PMC4477076; doi:10.1007/s00122-015-2527-8)
Supplement: Supplementary file 1 — Supplementary material 1 (PDF 97 kb) [file 122_2015_2527_MOESM1_ESM.pdf]

Online Resource 1

Article title: Comparative mapping of quantitative trait loci for Fusarium head blight resistance and anther retention in the winter wheat population Capo x Arina

Journal name: Theoretical and Applied Genetics

Authors: Maria Buerstmayr, Hermann Buerstmayr

Name, affiliation, and email of corresponding author:

Maria Buerstmayr
Department for Agrobiotechnology Tulln,
BOKU-University of Natural Resources and Life Sciences Vienna,
Konrad Lorenz Str. 20, Tulln 3430, Austria
e-mail: maria.buerstmayr@boku.ac.at

Online Resource 1 Means of the parents as well as mean, minimum and maximum values of the Capo x Arina RIL population for %FHB severity 26 days post inoculation, AUDPC and AUDPC transformed

		Parents		Population		
**Trait**	**Experiment**	**Arina**	**Capo**	**mean**	**min**	**max**
%FHB diseased spikelets[a]						
	2011	18.0	27.5	44.9	7.5	90.0
	2012	21.5	35.0	31.7	6.5	85.0
	2013	17.5	22.5	36.9	5.0	80.0
	overalll mean	19.0	28.3	37.8	6.7	80.0
AUDPC[b]						
	2011	120.2	182.6	274.9	36.0	698.5
	2012	164.6	224.8	241.3	26.4	709.0
	2013	154.4	264.7	436.8	62.1	1097.5
	overall mean	146.4	224.0	316.6	50.3	767.0
AUDPC (transformed data)[b,c]						
	2011	10.9	13.5	16.0	5.9	26.4
	2012	12.8	15.0	14.7	5.1	26.6
	2013	12.4	16.2	19.9	7.6	33.1
	overall mean	12.0	14.9	16.8	7.0	27.4

[a] %FHB diseased spikelets per plot 26 days post inoculation
[b] AUDPC = area under the disease progress curve
[c] square root transformed AUDPC data, used as measure of FHB severity for all further statistical analysis
