# Supplementary material for: Comparative mapping of quantitative trait loci for Fusarium head blight resistance and anther retention in the winter wheat population Capo × Arina
Source: Theor Appl Genet. 2015 May 16;128(8):1519–30. doi: 10.1007/s00122-015-2527-8 (PMC4477076; doi:10.1007/s00122-015-2527-8)
Supplement: Supplementary file 2 — Supplementary material 2 (PDF 35 kb) [file 122_2015_2527_MOESM2_ESM.pdf]

**Online Resource 2**

**Article title:** Comparative mapping of quantitative trait loci for Fusarium head blight resistance and anther retention in the winter wheat population Capo x Arina

**Journal name:** Theoretical and Applied Genetics

**Authors:** Maria Buerstmayr, Hermann Buerstmayr

**Name, affiliation, and email of corresponding author:**

Maria Buerstmayr
Department for Agrobiotechnology Tulln,
BOKU-University of Natural Resources and Life Sciences Vienna,
Konrad Lorenz Str. 20, Tulln 3430, Austria
e-mail: maria.buerstmayr@boku.ac.at

**Online resource 2** Spearman rank-correlation coefficients between traits and experiments

Spearman correlation coefficients between FHB severity, anther retention (AR%), plant height and flowering date of individual experiments and for the overall means

| | Overall mean | | | | | | 2011 | | | | 2012 | | | | | | 2013 | | | | | |
|---|---|---|---|---|---|---|---|---|---|---|---|---|---|---|---|---|---|---|---|---|---|---|
| | Plant height | | AR% | | Flowering date | | Plant height | | Flowering date | | Plant height | | AR% | | Flowering date | | Plant height | | AR% | | Flowering date | |
| | r | *p* | r | *p* | r | *p* | r | *p* | r | *p* | r | *p* | r | *p* | r | *p* | r | *p* | r | *p* | r | *p* |
| FHB severity[a] | -0.39 | <.0001 | 0.63 | <.0001 | 0.02 | 0.83 | -0.30 | <.0001 | -0.03 | 0.68 | -0.23 | 0.00 | 0.57 | <.0001 | 0.10 | 0.18 | -0.36 | <.0001 | 0.54 | <.0001 | 0.13 | 0.08 |
| Plant height | | | -0.14 | 0.06 | 0.18 | 0.02 | | | 0.04 | 0.62 | | | -0.18 | 0.02 | 0.29 | <.0001 | | | -0.06 | 0.46 | 0.17 | 0.03 |
| AR% | | | | | -0.08 | 0.29 | | | | | | | | | -0.01 | 0.87 | | | | | -0.06 | 0.41 |

[a] calculated from the transformed AUDPC data

Spearman correlation coefficients between experiments for FHB severity, plant height, flowering date and anther retention (AR%)

| | FHB severity[a] | | | | Plant height | | | | Flowering date | | | | AR% | |
|---|---|---|---|---|---|---|---|---|---|---|---|---|---|---|
| | 2012 | | 2013 | | 2012 | | 2013 | | 2012 | | 2013 | | 2013 | |
| | r | *p* | r | *p* | r | *p* | r | *p* | r | *p* | r | *p* | r | *p* |
| 2011 | 0.73 | <.0001 | 0.64 | <.0001 | 0.84 | <.0001 | 0.79 | <.0001 | 0.79 | <.0001 | 0.80 | <.0001 | | |
| 2012 | | | 0.60 | <.0001 | | | 0.82 | <.0001 | | | 0.73 | <.0001 | 0.77 | <.0001 |

[a] calculated from the transformed AUDPC data
