# Supplementary material for: Comparative mapping of quantitative trait loci for Fusarium head blight resistance and anther retention in the winter wheat population Capo × Arina
Source: Theor Appl Genet. 2015 May 16;128(8):1519–30. doi: 10.1007/s00122-015-2527-8 (PMC4477076; doi:10.1007/s00122-015-2527-8)
Supplement: Supplementary file 3 — Supplementary material 3 (PDF 123 kb) [file 122_2015_2527_MOESM3_ESM.pdf]

# Online Resource 3

**Article title:** Comparative mapping of quantitative trait loci for Fusarium head blight resistance and anther retention in the winter wheat population Capo x Arina

**Journal name:** Theoretical and Applied Genetics

**Authors:** Maria Buerstmayr, Hermann Buerstmayr

**Name, affiliation, and email of corresponding author:**

Maria Buerstmayr
Department for Agrobiotechnology Tulln,
BOKU-University of Natural Resources and Life Sciences Vienna,
Konrad Lorenz Str. 20, Tulln 3430, Austria
e-mail: maria.buerstmayr@boku.ac.at

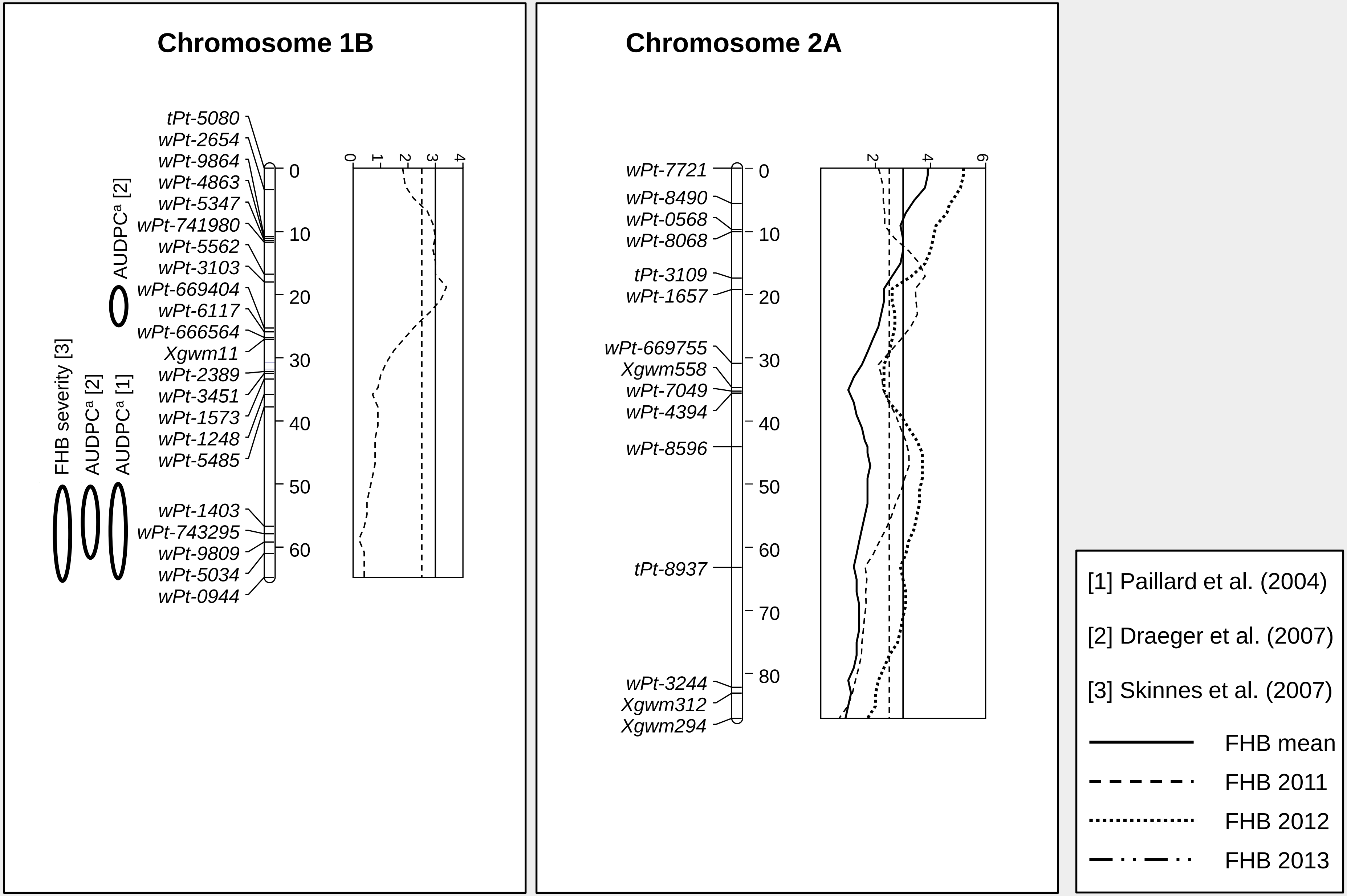


[a] FHB measured as area under the disease progress curve

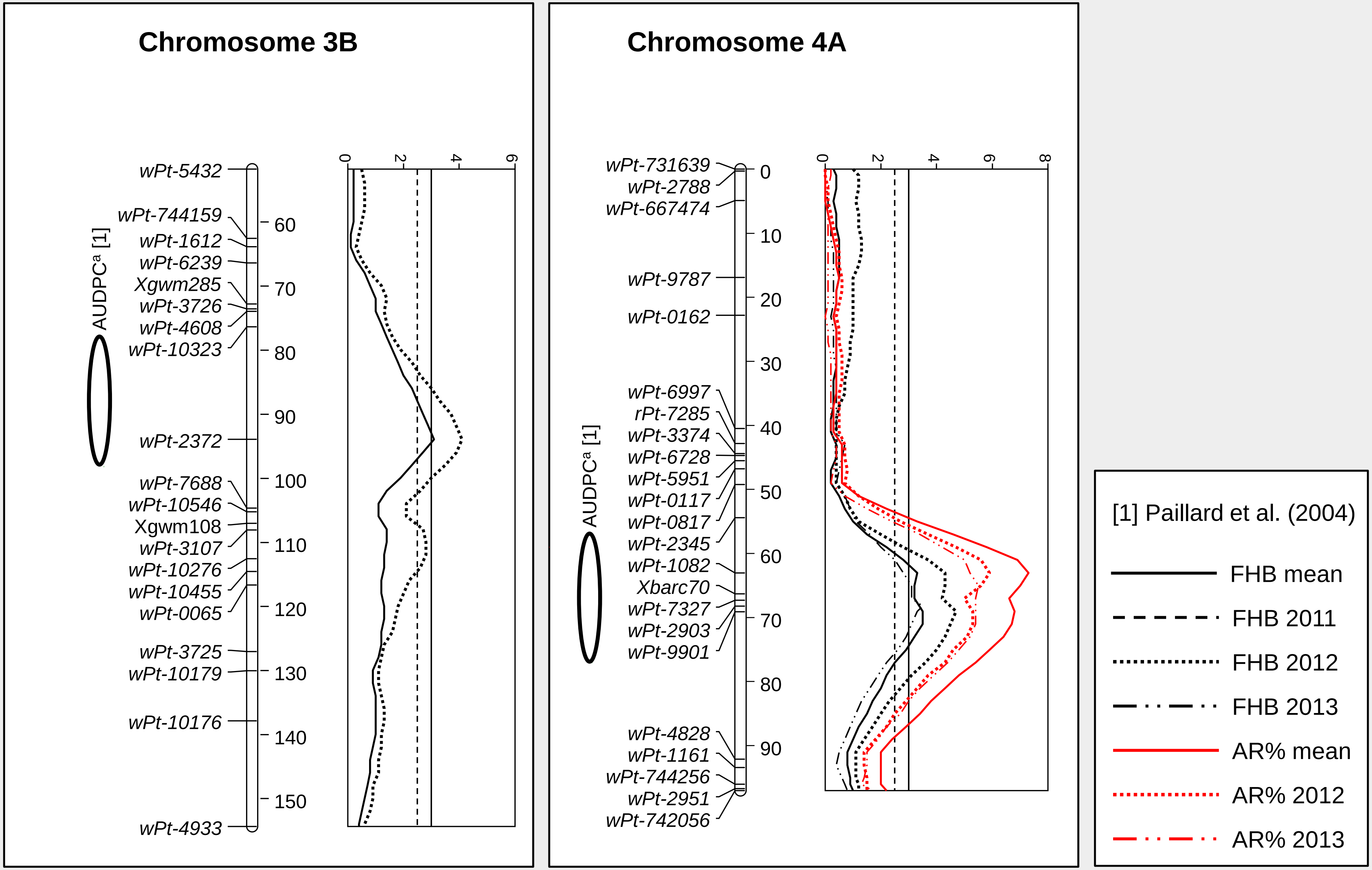


[a] FHB measured as area under the disease progress curve

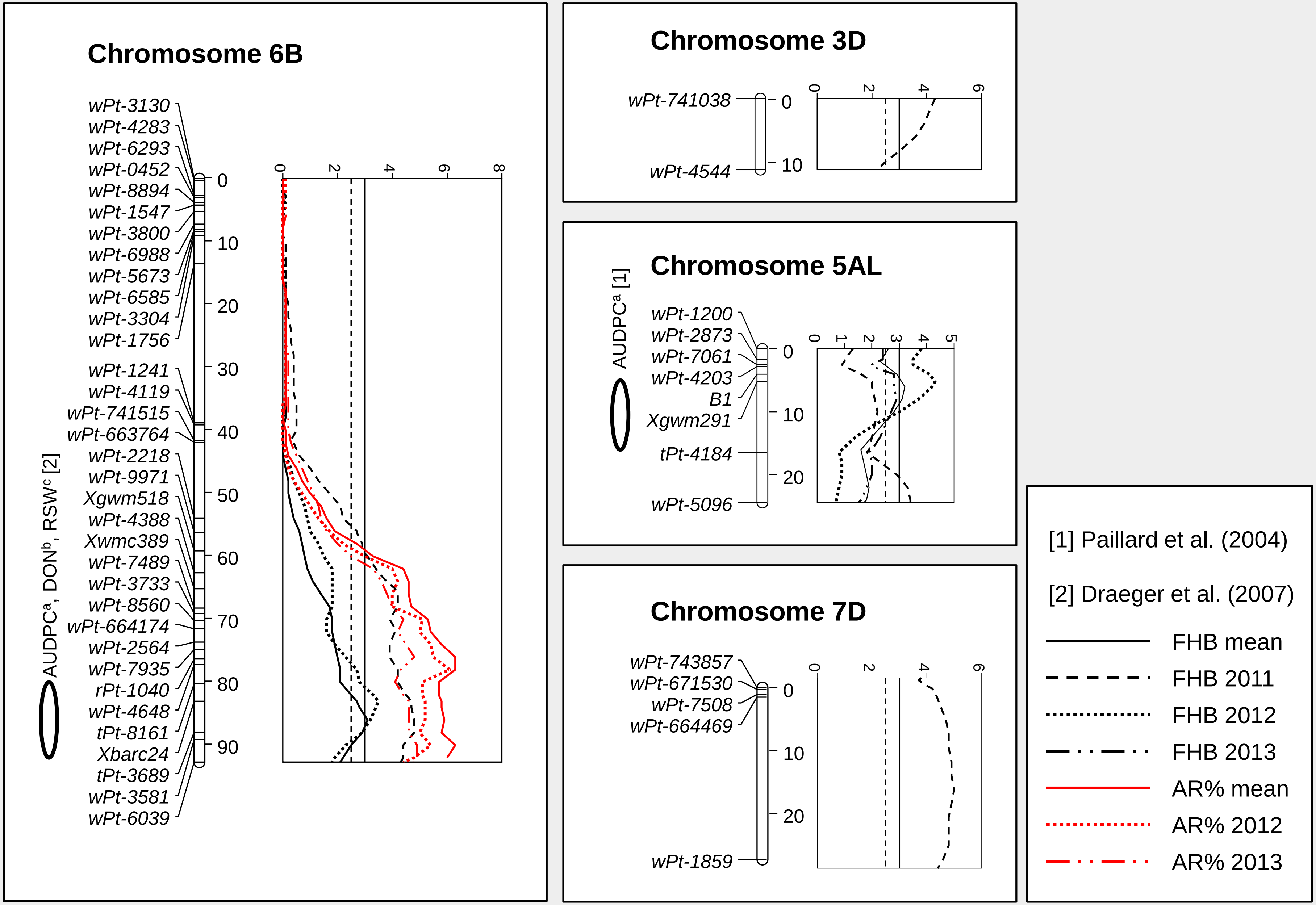


[a] FHB measured as area under the disease progress curve
[b] DON deoxynivalenol
[c] RSW relative spikelet weight

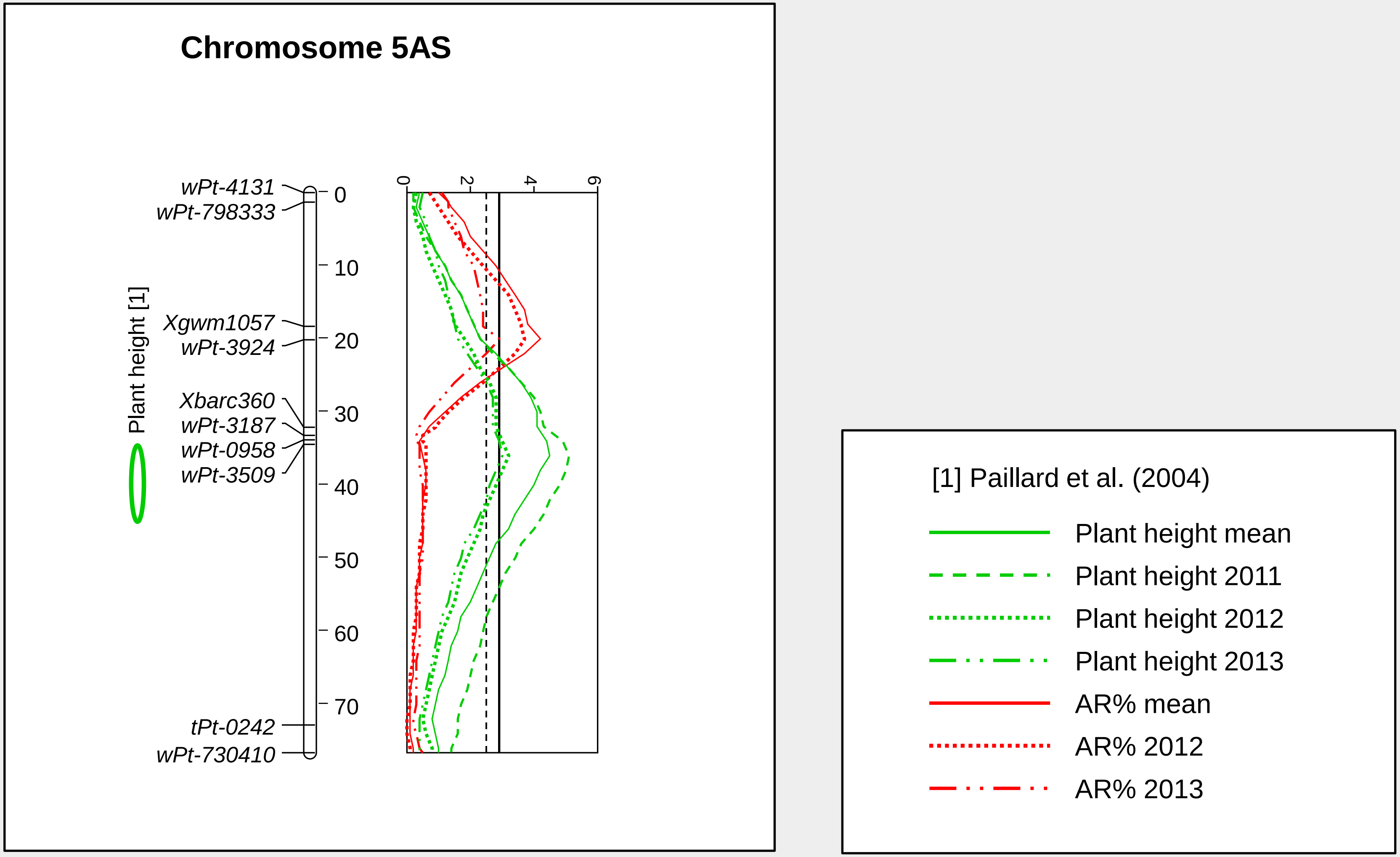


**Fig. S1** Linkage maps and QTL graphs determined by the MQM model. FHB QTL are based on the transformed AUDPC data. LOD profiles are given on the right. The dashed and solid lines represent the LOD 2.5 and 3 value, respectively. Elliptical bars on the left indicate the approximate QTL positions of QTL with the resistance allele contributed by cultivar Arina previously reported by Paillard et al. (2004) [1], Draeger et al. (2007) [2], or Skinnes et al. (2007) [3]. Numbers in brackets specify the corresponding publication.
